# Automatic extraction of candidate nomenclature terms using the doublet method

**DOI:** 10.1186/1472-6947-5-35

**Published:** 2005-10-18

**Authors:** Jules J Berman

**Affiliations:** 1 Cancer Diagnosis Program, National Cancer Institute, National Institutes of Health, Bethesda, MD, USA

## Abstract

**Background:**

New terminology continuously enters the biomedical literature. How can curators identify new terms that can be added to existing nomenclatures? The most direct method, and one that has served well, involves reading the current literature. The scholarly curator adds new terms as they are encountered. Present-day scholars are severely challenged by the enormous volume of biomedical literature. Curators of medical nomenclatures need computational assistance if they hope to keep their terminologies current. The purpose of this paper is to describe a method of rapidly extracting new, candidate terms from huge volumes of biomedical text. The resulting lists of terms can be quickly reviewed by curators and added to nomenclatures, if appropriate. The candidate term extractor uses a variation of the previously described doublet coding method. The algorithm, which operates on virtually any nomenclature, derives from the observation that most terms within a knowledge domain are composed entirely of word combinations found in other terms from the same knowledge domain. Terms can be expressed as sequences of overlapping word doublets that have more specific meaning than the individual words that compose the term. The algorithm parses through text, finding contiguous sequences of word doublets that are known to occur somewhere in the reference nomenclature. When a sequence of matching word doublets is encountered, it is compared with whole terms already included in the nomenclature. If the doublet sequence is not already in the nomenclature, it is extracted as a candidate new term. Candidate new terms can be reviewed by a curator to determine if they should be added to the nomenclature. An implementation of the algorithm is demonstrated, using a corpus of published abstracts obtained through the National Library of Medicine's PubMed query service and using "The developmental lineage classification and taxonomy of neoplasms" as a reference nomenclature.

**Results:**

A 31+ Megabyte corpus of pathology journal abstracts was parsed using the doublet extraction method. This corpus consisted of 4,289 records, each containing an abstract title. The total number of words included in the abstract titles was 50,547. New candidate terms for the nomenclature were automatically extracted from the titles of abstracts in the corpus. Total execution time on a desktop computer with CPU speed of 2.79 GHz was 2 seconds. The resulting output consisted of 313 new candidate terms, each consisting of concatenated doublets found in the reference nomenclature. Human review of the 313 candidate terms yielded a list of 285 terms approved by a curator. A final automatic extraction of duplicate terms yielded a final list of 222 new terms (71% of the original 313 extracted candidate terms) that could be added to the reference nomenclature.

**Conclusion:**

The doublet method for automatically extracting candidate nomenclature terms can be used to quickly find new terms from vast amounts of text. The method can be immediately adapted for virtually any text and any nomenclature. An implementation of the algorithm, in the Perl programming language, is provided with this article.

## Background

Samuel Johnson defines a lexicographer as a "harmless drudge" [1]. The drudgery of the lexicographer's tasks is beyond dispute. In the domain of medical nomenclatures, however, the harmlessness of the lexicographer is far from certain. The misuse of medical terminology can lead to medical errors, as indicated by the U.S. Joint Commission on Accreditation of Healthcare Organization's recent ban on certain common medical abbreviations [2]. This action was taken to reduce the occurrence of medication errors that result when non-standard abbreviations are misinterpreted. The U.S. Institute of Medicine has advocated standardized methods for collecting codified diagnostic data as a strategy for reducing medical errors [3].

Medical terminologies are replete with examples of minor term modification that can result in treatment error. One example is the "carcinoid tumor of appendix." Carcinoid tumors of the appendix are typically indolent. According to the U.S. National Cancer Institute, "Surgical resection is the standard curative modality. If the primary tumor is localized and resectable, 5-year survival rates are excellent" [4]. There is a variant of appendiceal carcinoid known as the goblet cell carcinoid of appendix. Someone uninitiated in tumor biology may infer that the goblet cell carcinoid is a morphologic variant of an indolent neoplasm undeserving of any special designation. Actually, the goblet cell carcinoid of appendix is a highly malignant tumor that has a completely different clinical course and a different recommended treatment than the carcinoid of appendix [5]. If a neoplasm nomenclature were to omit the term "goblet cell carcinoid of appendix", someone encountering the term in a pathology report may mistakenly code the subsumed term, "carcinoid of appendix." Alternately, if the curator of a nomenclature is unaware of the distinction between the two tumors, he or she may mistakenly assign the same code to both terms. In either case, mistaking a carcinoid of the appendix with a goblet cell carcinoid of appendix could result in harm to the patient.

Because modern nomenclatures are used to annotate medical data so that clinical information can be merged with heterogeneous data sources (e.g. tissue bank records, research datasets, epidemiologic databases), the duties of lexicographers have broadened to include a range of informatics activities. For this reason, the modern curator is involved in codifying terms (providing a unique identifier to a term and all its synonyms), and mapping terms between different nomenclatures. In the past, nomenclatures were recorded on paper documents. Brevity was appreciated, and rare lesions may have been neglected. Modern nomenclatures are stored electronically. With no barriers to the size of nomenclatures, there is no reason to exclude any used terms [6].

The purpose of this manuscript is to describe a simple method for extracting candidate new terms from any large corpus of text. The method uses the previously published doublet algorithm to compare connected word doublets in a medical text against a list of word doublets found in a nomenclature [7]. Text phrases composed of sequences of word doublets found in an existing nomenclature are candidate new nomenclature terms. This general method can be used with any text and any existing nomenclature. This method permits curators to continually enhance their nomenclatures with new terms, an essential activity needed to ensure the proper coding and annotation of biomedical data.

## Implementation

### Nomenclature

As a sample implementation, the developmental lineage classification and taxonomy of neoplasms, hereinafter called "the neoplasm taxonomy," was used. The neoplasm taxonomy has several properties that make it particularly suitable:

1. It is a free, open-access medical nomenclature.

2. It has been described in two prior open access publications [6,8]

3. New versions of the nomenclature are made available for public download by the Association for Pathology Informatics [9].

4. It is an easily parsed XML document, with every term appearing as a lower-case alphanumeric phrase.

5. It is intended to be a comprehensive listing of all items in a knowledge domain [i.e., names of neoplasms]

The XML version of the neoplasm taxonomy file is neocl.xml [see Additional file 1]. In this manuscript, the purpose of the taxonomy is to provide a listing of all names of neoplasms, with synonyms grouped under a common code number. The current version of the neoplasm taxonomy contains 149,192 unique names of neoplasms. In constructing the taxonomy, enormous effort was made to list every variant name for every known neoplasm of man. Variant names included different terms for the same concept and different ways of expressing an individual term (e.g. variations in word order).

### Input file

The input file was created by a PubMed query on "am j surg pathol [jo]", at the U.S. government website [10]. The query gathered all abstracts from the PubMed database that were published in the journal "American Journal of Surgical Pathology." The American Journal of Surgical Pathology is a popular repository for articles written to describing newly encountered neoplasms or distinctive variants of known neoplasms. The PubMed search produced 4,289 articles. These were downloaded to an external file using PubMed XML (i.e. by selecting "XML" from PubMed's "Display" list). The downloaded file has a length of over 31 Megabytes. Each of the 4,289 entries in the downloaded file contains metadata-tagged fields that include the PubMed identifier, the title of the article, the names of the authors, the text of the abstract, and other citation-related information. Titles of medical articles are terse summary statements that often contain the names of disease entities discussed within the article. Although this file is not included with this manuscript, anyone in the world with internet access can obtain a near-identical file by repeating the same PubMed query.

### Algorithms

The doublet method was described in a recent publication [7]. Its utility is derived in part from the observation that most medical terms are multi-word terms. In the neoplasm taxonomy, all but about 250 terms are multi-word terms. Unlike single words, which often have several different meanings, multi-word medical terms, with very rare exceptions, have a single, specific meaning.

Any multiword term can be constructed by a concatenation of overlapping doublets.

For example:

Serous borderline ovarian tumor -> ("serous borderline," "borderline ovarian," "ovarian tumor")

The doublets composing the multiword terms from a nomenclature can be combined into a list . The list of nomenclature doublets can be used to determine whether a fragment of text is composed from doublets included in the list.

#### Algorithm 1

The following algorithm determines those terms present in a nomenclature that are composed of doublets found in other terms within the nomenclature.

1. Collect all the doublets that occur in the entire nomenclature (i.e., accumulate a list of the doublets from every term in the nomenclature).

2. Number the occurrences of these doublets (i.e., count the number of times each doublet occurs anywhere in the nomenclature)

3. Advance through the nomenclature, term by term, decomposing the term into its doublets.

4. For each of the doublets that compose a term, retrieve the total number of occurrences of the doublet in the nomenclature.

5. Each doublet occurring in a term that has an occurrence number of 1 is unique to that term and indicates that the term is not composed entirely of doublets found in other terms in the nomenclature.

#### Algorithm 2

The following algorithm parses through text, extracting candidate term phrases:

1. Collect all the doublets that occur in the entire nomenclature (i.e., accumulate a list of the doublets from every term in the nomenclature).

2. Parse text (in this case individual abstract titles) into an ordered array of overlapping doublets (as per the example shown for the text string, "serous borderline ovarian tumor").

3. Compare each consecutive text doublet against the array of doublets from the nomenclature to determine whether the doublet exists somewhere in the nomenclature.

4. If the doublet from the text does not exist in the nomenclature, it can be deleted. If it exists in the nomenclature, it is concatenated with the following doublet if the following doublet exists in the nomenclature. Otherwise, it is deleted. This process continues, concatenating doublets that exist somewhere in the nomenclature. Extraneous leading words (the, in, of, with, and) and trailer words, (the, and, with, from, a) are automatically deleted from the final concatenated sequence. Final concatenated sequences of two or greater consecutive doublets that match to doublets from the nomenclature are saved as candidate terms.

### Software

#### 1. Programming Language

All code was written in Perl. Perl is an open source language that is bundled with the Unix and Linux operating systems. In the past decade, Perl has become very popular in the bioinformatics community. Perl interpreters are available at no cost and in versions suitable for virtually all operating systems. Perl can be obtained from the Comprehensive Perl Archives Network [11] or from Active State [12].

2. The Perl script doubuniq.pl implements algorithm 1 to locate terms in the nomenclature that contain any uniquely occurring doublets (i.e., terms that are not exclusively composed of doublets found in other terms from the same nomenclature) [see Additional file 2]. Only the classified terms of the nomenclature (a total of 126,756 terms) were used in this exercise to avoid formatting inconsistencies present in terms that are pending classification. The output of the Perl script is included as a supplemental file [see Additional file 3].

3. The Perl script getdoub.pl implements algorithm 2 to extract phrases from the text corpora composed of sequences of doublets included in the original nomenclature and exceeding 4 words in length [see Additional file 4]. The output file is tumoram.out [see Additional file 5].

## Results

### Analysis of doublet occurrences within terms included in the nomenclature

The current version of the neoplasm nomenclature contains 149,192 unique terms. Of these terms 126,756 terms are classified terms and are composed of at least two words (i.e., are doublets or greater in length). Of these 126,756 terms, all but 6,308 (4.97%) are composed entirely of doublets extracted from other terms in the reference nomenclature. This means that 95% of the classified terms from the nomenclature are formed entirely of doublet terms found in other terms from the same nomenclature. The Perl script and the output text are included as supplemental files [see Additional file 2] [see Additional file 3].

### Implementation of the candidate term extractor

The Perl script, getdoub.pl was run on a CPU with a processor speed of 2.79 GHz. The parsed corpus was a PubMed file in XML format that exceeded 31 Megabytes in length and contained 4,289 PubMed records. Each record contained an abstract title enclosed by a tag, <ArticleTitle>. The parser extracted the abstract titles demarcated by the XML tag, dropped all characters to lower-case, deleted all characters other than alphanumerics, spaces and hyphens, deleted the possessive suffix characterized by "'s" and converted plural words to singular words if the word ended in the suffix "oma" or consisted of the word "tumors" or "tumours". The total number of words contained in the 4,289 abstract titles was 50,547, and the total number of characters contained in the 4,289 abstract titles was 395,396. The total execution time to extract a list of candidate terms from the corpus was 2 seconds.

### Output file of candidate phrases

The output file produced by getdoub.pl is tumoram.out. It contains 313 candidate phrases each phrase followed by a copy of the original text from which the candidate term was extracted. The output is included as a supplemental file [see Additional file 5]. Each extracted candidate term consisted entirely of concatenated doublets found within the reference nomenclature.

In some cases, terms consisting of concatenated doublets were obviously not names of neoplasms, and a human expert in the knowledge domain would be needed to distinguish a real name of a neoplasm from a series of words that have no particular significance.

The curator read through all 313 phrases, looking for phrases that suggest a new concept. In this case, the curator looked for names of neoplasms. This step can only be performed by a domain expert. Because the exact candidate phrases are found in PubMed abstract titles, curators can obtain additional information on any candidate phrase by inserting the phrase into the PubMed query box [10]. In some cases, in-depth review of the literature containing the candidate phrase may suggest a slightly modified preferred term as a new entry to the nomenclature, but for the purposes of this manuscript, candidate phrases were not expanded or embellished with alternate words.

As example, consider line 100 from the output file [see Additional file 5]. The candidate term is: "synovial sarcoma of the prostate with t." This term is composed of the doublets, "synovial sarcoma", "sarcoma of", "of the", "the prostate", "prostate with", "with t". The full abstract title from which the term was extracted was: "SYNOVIAL SARCOMA OF THE PROSTATE WITH T(X;18)(P11.2;Q11.2)." The chromosomal marker designated in the abstract title is not picked up by the extracting Perl script because (X;18) is not part of a word doublet. For the purposes of this manuscript, the curator is only permitted to remove words from the beginning and end of the candidate phrase (not embellish the phrase). The curator shortens the term to "synovial sarcoma of the prostate". If that term is not included in the existing nomenclature, it can be added to the final list of new terms. In practice, a curator might wish to add two new terms to the nomenclature, "synovial sarcoma of the prostate" and "synovial sarcoma of the prostate with T(X;18) (P11.2;Q11.2)."

### Final list of new terms

The output file, tumoram.out contained 313 candidate terms [see Additional file 5]. Of these 313 terms, a human curator found 285 terms that represented names of neoplasms. These 285 terms were automatically evaluated to eliminate terms that were duplicated in the reference nomenclature or on duplicate lines from the output file. The final list of 222 new terms is shown here:

"acinic cell carcinoma of the lung, adenocarcinoma and large cell carcinoma of lung, adenomatous hyperplasia of the rete testis, adenosarcoma of the renal pelvis with heterologous elements, adenosarcoma of the uterus with sarcomatous overgrowth, adrenal cortical adenoma in the spinal canal, aggressive digital papillary adenoma and adenocarcinoma, anaplastic carcinoma of the lung, atypical burkitt lymphoma arising from follicular lymphoma, atypical duct hyperplasia of the breast, atypical fibrous histiocytoma of the skin, atypical polypoid adenomyoma of the uterus, b-cell lymphoma of mucosa-associated lymphoid tissue, benign cystic mesothelioma of the liver, benign epithelioid peripheral nerve sheath tumor of the soft tissues, benign mesenchymal tumor of lymph node, benign mesenchymal tumor of the uterus, benign schwannoma of the digestive tract, bile duct adenoma clear cell type, bizarre parosteal osteochondromatous proliferation of bone, borderline epithelial lesions of the breast, borderline papillary serous tumour of the fallopian tube, botryoid rhabdomyosarcoma of the biliary tract, breast tumor resembling the tall cell variant of papillary thyroid carcinoma, carcinoid tumor of the extrahepatic bile ducts, carcinosarcoma of the female genital tract, clear cell carcinoma of the gallbladder, clear cell carcinoma of the ovary, clear cell carcinoma of the skin, clear cell myomelanocytic tumor of the thigh, clear cell myomelanocytic tumor of the urinary bladder, clear cell sarcoma of soft tissues, clear cell sarcoma of the ileum, clear cell sugar tumor of the pancreas, clear cell tumor in the liver, clear cell tumor of the kidney, clear cell tumor of the lung, combined small cell and spindle cell carcinoma of the lung, composite follicular variant of papillary carcinoma and mucoepidermoid carcinoma of the thyroid, condylomata acuminata of the urinary bladder, congenital cystic adenomatoid malformation of the lung, cutaneous epidermotropic alveolar rhabdomyosarcoma, cutaneous follicle center b-cell lymphoma, cystadenofibroma of the ovary with epithelial atypia, cystic hamartoma of the renal pelvis, desmoplastic small cell tumor in the pancreas, desmoplastic small cell tumor of soft tissues, desmoplastic small round cell tumor of the pleura, diffuse mesothelioma of the genital tract and peritoneum, ductal adenoma of the breast, ductal carcinoma in situ in breast, ductal carcinoma in situ in salivary duct carcinoma, ductal carcinoma in situ of the female breast, dysplasia of the epididymis, endometrial stromal and smooth muscle tumor of the uterus, epithelial mesothelioma of the pleura, epithelial-stromal tumor of the seminal vesicle, epithelioid hemangioendothelioma of skin and soft tissues, epithelioid leiomyosarcoma of the skin and subcutaneous tissue, epithelioid mesothelioma of the pleura, ewing family of tumor, extragastrointestinal stromal tumor gastrointestinal stromal tumor of the soft tissue, extramammary paget disease of the oral mucosa, familial adenomatous polyposis associated with multiple endocrine neoplasia, foamy gland high-grade prostatic intraepithelial neoplasia, follicle center lymphoma of the ampulla of vater, follicular center-cell lymphoma with plasmacytic differentiation, follicular dendritic cell tumor of the liver, follicular dendritic cell tumor of the oral cavity, follicular thyroid carcinoma with clear cell change, gastric mucosa-associated lymphoid tissue, gastric neuroendocrine tumor in multiple endocrine neoplasia, gastrointestinal autonomic nerve tumor in the colon, germ cell neoplasms of the testis, giant cell tumor of soft tissues, glycogen-rich clear cell carcinoma of the breast, granular cell tumor of the lung, granulocytic sarcoma of the female genital tract, hematopoietic tumor in chronic myeloproliferative disorders, high-grade dysplasia in a colonic adenoma, histiocytic lymphoma of the central nervous system, histiocytic sarcoma involving the central nervous system, hyalinizing trabecular adenoma and papillary carcinoma of the thyroid gland, hyalinizing trabecular tumor of the thyroid gland, hyperplasia of interstitial cells of cajal, infiltrating colloid carcinoma of the pancreas, inflammatory fibroid polyp of the stomach, intestinal metaplasia of the distal esophagus, intraductal carcinoma of the oral cavity, juvenile granulosa cell tumor of the ovary, keratinizing squamous cell cancer of the cervix, langerhans cell granulomatosis of the thymus, large cell lymphoma of the mediastinum, lobular carcinoma in situ of the female breast, low grade b-cell lymphoma of mucosa associated lymphoid tissue, low malignant potential tumor of the ovary, low-grade b-cell lymphoma of mucosa-associated lymphoid tissue, low-grade endometrial stromal sarcoma, lymphoid hyperplasia of the gastrointestinal tract, lymphoma of the gastrointestinal tract, malignant epithelioid hemangioendothelioma of the liver, malignant epithelioid schwannoma, malignant fibrous histiocytoma of the extremities, malignant fibrous tumor of the pleura, malignant giant cell tumor of the tendon sheaths, malignant lymphoma of the mucosa-associated lymphoid tissue, malignant lymphoma of the small bowel, malignant lymphoma of the upper aerodigestive tract, malignant melanoma of the esophagus, malignant melanoma of the urethra, malignant mesonephric tumor of the female genital tract, malignant mesothelioma of the tunica vaginalis, malignant neuroendocrine tumor of the jejunum with osteoclast-like giant cells, malignant ovarian tumor with osteoclast-like giant cells, malignant peripheral nerve sheath tumor of infancy, malignant small cell tumor of thoracopulmonary region, marginal zone b-cell lymphoma in children and young adults, marginal zone b-cell lymphoma of the salivary gland arising in chronic sclerosing sialadenitis, melanocytic neoplasms of the central nervous, metaplasia of the renal collecting ducts, microinvasive squamous carcinoma of the esophagus, micropapillary variant of transitional cell carcinoma of the urinary bladder, mixed endometrial stromal and smooth muscle tumor of the uterus, mucin-secreting adenocarcinoma of the prostate with neuroendocrine differentiation, mucinous adenocarcinoma of the prostate gland, mucinous borderline tumor of the ovary mucinous cystic tumor of the ovary, multiple lymphomatous polyposis of the gastrointestinal tract, myoepithelial carcinoma of the salivary glands, neuroendocrine merkel cell carcinoma, nonsmall cell carcinoma of the lung, oat cell carcinoma of the kidney, oncocytic mucoepidermoid carcinoma of the salivary glands, ovarian sertoli cell tumor with retiform and heterologous elements, papillary carcinoma of the bladder, papillary cystic tumor of the pancreas, papillary transitional cell carcinoma of the renal pelvis, papillary urothelial neoplasms of low malignant potential, pediatric soft tissue tumor, peripheral nerve sheath tumor of the adrenal, peritoneal serous micropapillomatosis of low malignant potential serous borderline tumor of the peritoneum, perivascular epithelioid clear cell family of tumor, peutz-jeghers syndrome with adenoma and adenocarcinoma, plasma cell granuloma of the lung, plasmablastic lymphoma of the oral cavity, pleomorphic adenoma mixed tumor of the major salivary glands, pleomorphic hyalinizing angiectatic tumor of soft parts, pleomorphic large cell lymphoma of the mediastinum, pleomorphic soft tissue myogenic sarcoma of adulthood, polycystic adenosis of major salivary glands, precursor of uterine papillary serous carcinoma, primary embryonal rhabdomyosarcoma of long bone, primary hepatic non-hodgkin lymphoma in childhood, primary lung carcinoma with signet-ring cell carcinoma, primary lymphoma of the small intestine, primary malignant lymphoma of the cauda equina, primary malignant melanoma of the anterior mediastinum, primary mixed adenocarcinoma and small cell carcinoma of the appendix, primary mucinous carcinoma of the skin, primary mucinous tumor of the ovary, primary neuroendocrine merkel cell carcinoma of the skin, primary ovarian small cell carcinoma, primary ovarian squamous cell carcinoma, primary squamous cell carcinoma of the ovary, primary synovial sarcoma of the kidney, primary tumor of the exocrine pancreas, pulmonary carcinoma with pleomorphic sarcomatoid or sarcomatous elements, pulmonary malignant lymphoma of mucosa-associated lymphoid tissue, reactive lymphoid hyperplasia of the spleen, renal angiomyolipoma with epithelioid sarcomatous transformation, renal cell carcinoma in children and young adults, renal cell carcinoma in tuberous sclerosis, reticulum cell neoplasms of lymph nodes, rhabdoid tumor of the gastrointestinal tract, round cell tumor of the tibia, sclerosing mucoepidermoid thyroid carcinoma with eosinophilia, serous borderline tumor of the peritoneum, serous borderline tumor of the peritoneum, signet-ring cell lymphoma of t-cell origin, small cell anaplastic carcinoma of the lung, small cell sweat gland carcinoma, small cell sweat gland carcinoma in childhood, small cell tumor of the lung, small cleaved cell lymphoma in bone marrow, small lymphocytic lymphoma leukemia, smooth muscle tumor of the gastrointestinal tract, smooth muscle tumor of the liver, smooth muscle tumor of the ovary, soft tissue lymphoma of the extremities, solid and cystic papillary epithelial neoplasm of the pancreas, solitary fibrous tumor of soft tissue, solitary fibrous tumor of the kidney, solitary fibrous tumor of the liver, solitary fibrous tumor of the mediastinum, solitary fibrous tumor of the nasal cavity, solitary fibrous tumor of the orbit, solitary fibrous tumor of the spinal cord, solitary fibrous tumor of the thyroid gland, solitary fibrous tumor of the upper respiratory tract, spindle cell and epithelioid cell nevi with atypia, spindle cell neoplasms of lymph nodes, spindle cell tumor of the pleura, spindle-cell carcinoid tumor of the lung, spindle-cell carcinoma of the upper aerodigestive tract, squamous cell carcinoma of the palatine tonsil, squamous cell carcinoma of the upper aerodigestive tract, stromal neoplasms of the gastrointestinal tract, systemic mast cell disease involving the bone marrow, t-cell histiocyte-rich large b-cell lymphoma of the spleen, t-cell lymphoma in the small intestine, t-cell-rich b-cell lymphoma of the spleen, thymic squamous cell carcinoma associated with mixed type thymoma, transitional cell carcinoma with rhabdoid features, transitional cell metaplasia of the uterine cervix, transitional cell tumor of the pituitary, urothelial carcinoma of the renal pelvis, urothelial transitional cell neoplasms of the urinary bladder, variant of malignant peripheral nerve sheath tumor, vascular hamartoma of the small bowel, well-differentiated neuroendocrine tumor, well-differentiated papillary mesothelioma of the pleura, yolk sac tumor of the anterior mediastinum."

Examination of the terms indicates that the majority of terms are either very rare and obscure tumors, unusual variants of common tumors, or just unusual terms for common and uncommon tumors. Pathologists reading this list may agree that the tumors from this list include clinically distinct and significant subclasses of tumors and would be welcome additions to a truly comprehensive nomenclature of neoplastic entities.

## Discussion

Adding terms to an existing vocabulary is best done by reading the current literature in the knowledge domain of the nomenclature, and transcribing new terms when they are encountered. It is difficult to imagine any automatic process that can replace this scholarly pursuit. Terms encountered while reading a scientific text appear in a structured context that often defines the term, clarifies the relationship of the new term to related terms, and sometimes provides sufficient information to classify the new term within a structured taxonomic hierarchy.

Sadly, it is impossible for curators to read all of the biomedical literature pertaining to a nomenclature's domain. The purpose of the doublet phrase extractor is to parse through any corpus of text, extracting phrases that may contain new nomenclature terms. The phrases are chosen to meet two criteria: 1) they are composed of word doublets that are contained in an existing nomenclature, and 2) the matched phrases do not already occur in the nomenclature. The doublet extractor works fast to produce a neat list of candidate phrases that can be conveniently reviewed by a curator. In the case described herein, a 31+ MByte corpus was extracted in 2 seconds, to produce 313 candidate phrases. From the candidate phrases, the curator found 222 phrases that could be added to the reference nomenclature (30 minutes of human effort).

Because the final term list was extracted from PubMed abstract titles, the contained terms have two important properties:

1. The terms have whatever legitimacy publication confers. Nobody can say that the extracted terms are confabulated or never actually used.

2. The terms can be searched and found through a PubMed search. The articles that contain the terms are likely to describe or define the terms.

### Limitations of the output

New terms that are not composed of doublets found in pre-existing nomenclature terms will be missed by the doublet extraction method. This would certainly apply to most new eponymous terms, new terms extracted from a foreign language, new terms using variant orthography, or newly invented words (e.g. theragnostics, glycomics, nanoscope). In the realm of neoplasms, it would be difficult to create a new term that is not partially composed of older terms.

If the extraction software used a vocabulary that already contained every term in the knowledge domain, then there would be very few candidate terms extracted (because parsed terms already included in the nomenclature are immediately rejected). In addition, any candidate terms extracted from the corpus would always be rejected by the human curator (0% precision). This is because the nomenclature would be complete, and any candidate terms must be false terms.

If the extraction software used a text corpus that consisted exclusively of valid, new nomenclature terms that should be added to an incomplete vocabulary, then all of the extracted candidate terms would be valid new terms (100% precision). If the extraction used a text that contained no valid terms from the knowledge domain, then every extracted candidate term would be an invalid term (0% precision).

This is a problem: the effectiveness of the software is dependent on the completeness of the source vocabulary and on the presence of valid new terms existing in the text corpus. In addition, the suitability of candidate terms for inclusion in the vocabulary will ultimately depend on subjective decisions reached by the human curator.

For this manuscript, the author chose to include the extractor's output as a supplemental file [see Additional file 5], so that interested readers can form their own judgments regarding the value of the software. In addition, the availability of a public set of files (software implementation, nomenclature and text corpus) will facilitate future efforts to compare improved versions of the extraction algorithm with this first version.

### Characteristics of the approach

Recently, Krauthammer and Nenadic reviewed methods for identifying nomenclature terms within text [14]. In all cases, the reviewed methods were evaluated based on published measurements of precision and recall. There was no common procedure for determining how the text would be manually coded. Manually coded text is used to measure the precision and recall, and if the rules for manual coding differ from study to study, so would the published performance measurements [15]. The speed of the different methods was not compared, and there was no common text corpus or nomenclature for the different studies. Most of the discussed methods were published in the Proceedings of various data mining workshops, and the source code, source vocabularies, and output files were not made available to the public. Because the source code for most of these methods is not accessible, it is difficult to determine the degree in which the methodology is vocabulary-dependent and whether the methods have generalized utility. For these reasons, it is impossible to state whether the doublet method is superior to previously published methods [16-19].

It is the perception of the author that the field of medical informatics suffers from a lack of simple and generalized methods that can be easily tested in various use cases or compared with alternate or new methods using shared corpora. In their review of methods for automatically identifying medical terms contained in text, Krauthammer and Nenadic commented that few terminologically tagged biomedical corpora are available [14], and this makes it impossible to compare different informatics methods.

This manuscript describes a method for extracting candidate terms from a corpus of text. It uses the doublet method previously described [7]. The doublet method has similarities to data retrieval techniques using bi-grams and word-pairs [20]. The method is fast, convenient, and suits the purposes of the author, who curates the neoplasm taxonomy. The method requires substantial human involvement to winnow down the list of candidate terms into a final collection of well-formed new nomenclature terms. The methodology has the following features:

1. The method will work with any nomenclature and with any plain-text file

2. The implementation is provided with the details of operation, allowing others to repeat the study.

3. The source code is platform-independent and is made freely available to the public. This permits other laboratories to modify the program and publish an improved version of this documented prototype.

4. The output files are provided, permitting others to review the results from this study or to compare the results with the results obtained with other methods.

## Conclusion

Almost all terms in medical nomenclatures can be formed by combining word doublets derived from other terms in the same nomenclature. By collecting all the word doublets found in a nomenclature, it is possible to quickly parse through enormous tracts of text, extracting phrases composed of sequences of these doublets. From these extracted candidate terms, a curator can select new terms. The doublet method is a novel approach to term extraction that curators can use to augment medical nomenclatures.

## Availability and requirements

The provided supplementary scripts are short programs written in Perl. Perl is a freely available open source programming language. Perl interpreters for virtually any operating system are available from several sites on the web [11,12]. These sites have links to rich sources of online information on the Perl language. The scripts require an external file of PubMed XML text. The scripts can be easily modified to accept any plain-text external file. The scripts require the neoplasm taxonomy [see Additional file 1], but can be modified to accept any parsable nomenclature that contains listed plain-text terms.

The Perl scripts provided with this manuscript will work without modification using the current version of the neoplasm taxonomy [see Additional file 1]. New versions of the neoplasm taxonomy are posted at the Association for Pathology Informatics website [9].

## Competing interests

The author(s) declare that they have no competing interests.

## Authors' contributions

The work expresses the opinion of the author and does not represent policy of the U.S. government.

## Pre-publication history

The pre-publication history for this paper can be accessed here:



## Supplementary Material

Additional file 1 Neoclxml.gz is the compressed version of neocl.xml, the XML-format for the developmental lineage classification and taxonomy of neoplasms. This version of neocl.xml supercedes prior published versions [6,7]. Because neocl.xml exceeds 9 Megabytes when uncompressed, a gzipped version of the file is provided (neoclxml.gz). After downloading from the biomedcentral site, the filename should be provided with a .gz suffix (if absent from the filename as downloaded). After decompressing the file, the file shoud be renamed "neocl.xml". The file can be viewed on current web browsers, but experience has shown that many browsers lack sufficient memory to display the entire file. Otherwise, the file can be viewed on a wordprocessor or an ascii editor. Click here for file

Additional file 2 Doubuniq.pl is a Perl script that parses the reference nomenclature (neocl.xml) and ouputs a list of terms that contain one or more unique doublets (i.e., terms that contain a doublet that is not found in any other term from the same nomenclature). Click here for file

Additional file 3 Doubuniq.txt is the output file of doubuniq.pl, and consists of 6,305 terms from the reference nomenclature (neocl.xml) that contain one or more unique doublets (i.e., terms that contain a doublet that is not found in any other term from the same nomenclature). Click here for file

Additional file 4 Getdoub.pl is a Perl script that implements the doublet method for automatic extraction of candidate nomenclature terms. It requires an external plain-text corpus and a reference nomenclature. Click here for file

Additional file 5 Tumoram.out is a plain-text file containing the output of the getdoub.pl, the Perl script that extracts terms consisting of concatenated doublets found in the reference nomenclature. Click here for file
